# Future diets in India: A systematic review of food consumption projection studies

**DOI:** 10.1016/j.gfs.2019.05.006

**Published:** 2019-12

**Authors:** Carmelia Alae-Carew, Frances A. Bird, Samira Choudhury, Francesca Harris, Lukasz Aleksandrowicz, James Milner, Edward JM. Joy, Sutapa Agrawal, Alan D. Dangour, Rosemary Green

**Affiliations:** a Department of Population Health, London School of Hygiene & Tropical Medicine, London, WC1E 7HT, UK; b Centre for Development, Environment and Policy, School of Oriental & African Studies, London, WC1H 0XG, UK; c Department of Public Health, Environments & Society, London School of Hygiene & Tropical Medicine, London, WC1H 9SH, UK; d Centre for Chronic Conditions and Injuries, Public Health Foundation of India, Gurgaon, Haryana, India

**Keywords:** India, Future diets, Food consumption, Food projections

## Abstract

Against a backdrop of a rapidly changing food system and a growing population, characterisation of likely future diets in India can help to inform agriculture and health policies. We systematically searched six published literature databases and grey literature repositories up to January 2018 for studies projecting the consumption of foods in India to time points beyond 2018. The 11 identified studies reported on nine foods up to 2050: the available evidence suggests projected increases in per capita consumption of vegetables, fruit and dairy products, and little projected change in cereal (rice and wheat) and pulse consumption. Meat consumption is projected to remain low. Understanding and mitigating the impacts of projected dietary changes in India is important to protect public health and the environment.

## Highlights

• First systematic review of future projected dietary changes in India.
• Per capita rice, wheat and pulse consumption projected to remain constant.
• Per capita sugar, dairy, vegetables and fruit consumption projected to increase.
• Projected shifts in future Indian diets likely to have health and environmental impacts.

## 1 Introduction

Diets in India are changing and in recent decades there has been a decline in the consumption of some cereals such as millets, while the consumption of salt, oils and animal products have increased (Misra et al., 2011). These dietary changes are likely to have contributed in part to an increased burden of non-communicable diseases (NCDs) in India, a trend seen across many low- and middle-income countries as populations become increasingly urban and incomes rise (World Health Organization, 2003). An estimated 61% of deaths in India were attributable to NCDs in 2017 (World Health Organization, 2017), while the prevalence of stunting (short height for age) amongst children under 5 years old remains extremely high at almost 40% (UNICEF, 2017), indicative of the co-existence of overweight and obesity with under-nutrition (the “dual burden”). Additionally, two-thirds of the Indian population are estimated to be micronutrient deficient, with their diets failing to provide recommended levels of minerals and vitamins such as iron and vitamin A that are typically found in the Indian diet in pulses, coarse cereals, and dark green leafy vegetables (Rao et al., 2018). Addressing this diet-related burden of disease in India remains a pressing need.

Changing dietary patterns may also have impacts on environmental parameters (Foley et al., 2005). The agriculture sector accounted for 17.6% of India's greenhouse gas (GHG) emissions in 2007 (Indian Network for Climat, 2010), and due to its large population, India is already the 4th largest emitter of GHGs in the world. Per capita GHG emissions associated with current dietary patterns in India are relatively low compared with that of other countries largely due to low consumption of animal products (Green et al., 2018), but future dietary changes in conjunction with continuing population growth (United Nations. Populatio, 2017) could make it hard for India to meet its targets of reducing GHG emissions intensity by 33–35% below 2005 levels by 2030 (Government of India, 2015). The agriculture sector is also a major user of ground and surface water (FAO AQUASTAT, 2016) and recent changes in dietary patterns in India are linked to increased demand for irrigation water (Harris et al., 2017). This poses an additional issue for environmental sustainability as irrigation water for agricultural use is increasingly being drawn from rapidly depleting groundwater resources (Rodell et al., 2009). Current and future trends in Indian diets therefore have potential implications for health, GHG emissions, ground and surface water availability, and potentially several other environmental factors.

Projecting future diets can provide useful information for national and international organisations involved in designing and delivering food and agriculture policy. These projections also enable researchers and policy makers to identify future health, nutritional and environmental impacts of projected dietary changes and inform possible mitigation strategies. The study of dietary projections is interdisciplinary and there is currently no ‘gold standard’ approach, nor a consensus on reporting standards. There has to-date been no systematic review of the dietary projections undertaken for India. We therefore performed a systematic review of studies that have projected future diets in India; we aimed to identify trends in projected future intake of foods, with consideration of study methodology and reporting quality.

## 2 Methods

### 2.1 Search strategy

This review follows the Preferred Reporting Items for Systematic Reviews and Meta-Analyses (PRISMA) guidelines (Liberati et al., 2009). A literature search of peer-reviewed and grey literature was performed to identify all studies that modelled future consumption of one or more food items or groups in India to at least one time point in the future. All studies were included from the beginning of the databases until the date of search (31st January 2018).

For published literature, six electronic databases were searched systematically, including the major interdisciplinary databases, and databases related to food and health: EMBASE, Global Health, MEDLINE, PubMed, Scopus and ISS Web of Science core collection. We also searched Google Scholar, the CGIAR research platform, and the repositories of organisations named in identified studies; namely the United Nations Food and Agriculture Organization (FAO), Indian Council of Agricultural Research National Institute of Agricultural Economics and Policy Research (ICAR-NIAP), the International Food Policy Research Institute (IFPRI), and the International Water Management Institute (IWMI). Citation lists of papers identified for inclusion and relevant review articles were hand-searched to identify additional studies. Authors were contacted if the full text was not found (3 authors contacted; 2 responses; 0 articles provided). The search strategy was developed initially in PubMed with the same search terms used with adjustments as needed for other databases. The search terms are summarised in Table 1 and the full search strategy for each database is detailed in supplementary material S1.

**Table 1:** Search terms used in electronic database search.

Food-related	Time-related (near to diet*)	Location-related
Food	Project*	India*
Diet	Future	Asia*
Nutri*	Trend*	“South Asia*”
Consum*	Transition*	Global
Nourish*	Change*	
	Predict*	
	Forecast*	
	Prognos*	

### 2.2 Selection criteria & data extraction

Titles were screened by a single reviewer (CC) for relevance. Abstracts were screened in duplicate (CC, FB) and consensus on any discrepancies reached through discussion with a third reviewer (FH). The population outcome (P0) framework (James et al., 2016) was followed to develop the inclusion criteria for studies to be selected for the review:

Population:

• The studies projected direct (human only) consumption in India

Outcome:

• Projections were of one or more food items or food groups
• The projections presented were the original work of the author(s)
• Consumption was projected to at least one time point in the future beyond the year in which this review was conducted (2018)
• Published in English

Relevant data were extracted by a single reviewer (CC) from the identified studies into a database, and all data were checked against the original studies by a second reviewer (SC). Extracted data were as follows: study authors and publication year; food consumption data source; projection model method; assumptions and variables; GDP growth rate scenarios; food groups or items; baseline year & values; and projection years & values.

### 2.3 Study reporting quality

There are no existing criteria for evaluating quality of dietary projection modelling studies. Reporting quality assessment parameters were developed by two reviewers (CC, FB) using the Authority, Accuracy, Coverage, Objectivity, Date, Significance (AACODS) grey literature checklist (Tyndall, 2010) and a set of quality criteria used previously for critiquing dietary simulation models (Grieger et al., 2017). Six parameters relating to the clear description of methodology and reporting of results were used to assess study reporting quality, one point was given for each criterion and quality scores ranged from 0 to a maximum of 6 (supplementary material S2).

### 2.4 Data analysis

The methods and data sources used in included projection studies were highly heterogeneous and quantitative synthesis was not possible. A narrative synthesis approach was used and extracted data from each study was tabulated. Baseline and projected consumption estimates were presented graphically for food groups reported in two or more studies. For studies that reported projections under different scenarios (typically several estimates of economic growth over the projection period) a single “most conservative” estimate was presented graphically to avoid over representation of individual studies. Dietary consumption values reported as kg/year were converted to kg/capita/month using the baseline and projected population estimates reported by study authors. Missing baseline population estimates were obtained from United Nations estimates for the appropriate year (United Nations. Populatio, 2017). Dietary consumption values reported in one paper as kcal/capita (Alexandratos and Bruinsma, 2012) could not be converted to kg/capita/month due to the aggregation by the study authors of food items into food groups.

## 3 Results

**Fig. 1:**
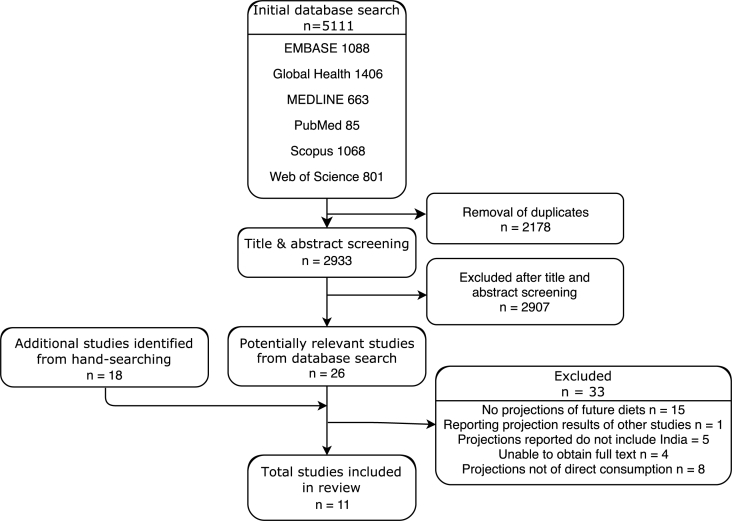
PRISMA chart showing the numbers of studies at each stage of the search.

The initial database search identified 5,111 studies. After removal of duplicates and screening of titles and abstracts, 26 studies remained. Hand-searching of review papers and reference lists identified a further 18 studies for full text screening. Of the 44 potentially relevant studies, 33 were excluded during full text screening (full texts of 4 potentially relevant studies could not be obtained despite multiple attempts). A total of 11 papers reporting projections of direct food consumption (i.e. food consumed by humans only) were included in the review (Fig. 1). Seven excluded studies predicted trends in total food consumption (i.e. food consumed by humans in addition to food used as agricultural seed, animal feed, food for industrial use and waste), and one study did not state whether projections related to direct or total food consumption.

### 3.1 Data sources and projection methods

The eleven included studies varied substantially in data sources, timescales, level of aggregation and projection models adopted (Table 2). Studies used different baseline years and projected for between 10 to 50 years, to future time points between 2020 and 2050; most studies projected consumption to between 2020 and 2026, two studies projected to 2050. In seven of the eleven included studies, consumer expenditure data from the National Sample Survey Office (NSSO) large-scale nationally representative Indian household surveys were used to determine food demand at baseline (Amarasinghe et al., 2007; Bhalla et al., 1999; Chand, 2007; Dastagiri, 2004; Dyson and Hanchate, 2000; Ganesh-Kumar et al., 2012; Kumar et al., 2009). Other studies obtained baseline data from: household consumer expenditure from the World Bank combined with United Nations Food and Agriculture Organisation (FAO) food balance sheets (n = 1) (Alexandratos and Bruinsma, 2012); United States Department of Agriculture (USDA) Production, Supply and Distribution data (PSD) (n = 1) (Carriquiry et al., 2010); a combination of Organisation for Economic Co-operation and Development (OECD) and FAO databases (n = 1) (OECD-FAO, 2017); and FAO food balance sheets (n = 1) (Rosegrant et al., 1999). Five studies disaggregated their results into rural and urban populations (Amarasinghe et al., 2007; Chand, 2007; Dastagiri, 2004; Dyson and Hanchate, 2000; Kumar et al., 2009), and one study at regional and state level (Dyson and Hanchate, 2000).

**Table 2:** Overview of characteristics of studies included in the systematic review.

Author & Year	Food Consumption Data Source	Disaggregation	Projection Model Method	Assumptions	Variables	Food Groups	Baseline Year	GDP Scenarios	Projection Years
Alexandratos and Bruinsma, 2012 (FAO) (Alexandratos and Bruinsma, 2012)	FBS & HHCE from World Bank (2005	No	Per capita calorie intake derived from consumer expenditure projections from 62 developing countries. Demand for each food group derived from proportional share of total calories			Cereals Milk & dairy Sugar (raw) Vegetable oils Meat	2005/07	4.4%	2050
Amarasinghe et al., 2007 (Amarasinghe et al., 2007)	48th, 50th & 55th round NSSO consumer expenditure	Rural/urban	Future trends in calorie intake are based on average global consumption patterns of 85 countries over 3 years. K-means cluster analysis used to capture variation in coefficients arising from taste and cultural differences		Per capita income growth Urbanisation Income elasticities	Rice Wheat Maize Other cereals Pulses Oil crops Roots & tubers Vegetables Fruits Sugar Disaggregated: Beef, pork & mutton Milk products Eggs Poultry Fish	2000	Not stated	2025 2050
Bhalla et al., 1999 (Bhalla et al., 1999)	50th round NSSO consumer expenditure	No	Demand projections based on variables listed	No change in income expenditures since 1993 Livestock production levels remain as per 1993	Population growth Urbanisation Expenditure elasticities Per capita income Changes in taste	Cereals Meat & eggs Milk & milk products	1993	2% 3.7% 6%	2020
Carriquiry et al., 2010 (FAPRI) (Carriquiry et al., 2010)	USDA PSD (supplemented by FAO database)	No	FAPRI World agricultural outlook model using IHS Global Insight macroeconomic forecasts	Average weather patterns Existing farm policy unchanged Existing trade agreements unchanged Existing custom unions unchanged	GDP growth GDP deflator growth Population growth	Wheat Rice Corn Sorghum Vegetable oil Sugar Beef & veal Poultry Dairy	2009/10	Not stated	2019/2020
Chand, 2007 (Chand, 2007)	61st round NSSO consumer expenditure	Rural/urban	Demand projections based on variables listed	Indian Economy growth rate 7.57% to 2012, 7.81% thereafter Urban income growth rate 3 times that of rural population	Income elasticity Per capita income growth Change in preferences Population growth Urbanization	Rice Wheat Coarse cereals Pulses Total cereals Foodgrains	2004/05	9%	2020/21
Dastagiri, 2004 (Dastagiri, 2004)	50th round NSSO consumer expenditure	Rural/urban	Generalized Least Squares procedure	Inequality in rural/urban expenditure remains as 1993/94	Expenditure elasticities Population growth Per capita income growth Price elasticities Urbanisation	Milk Mutton & goat meat Beef & buffalo meat Chicken Egg	1993	4% 5% 7%	2020
Dyson and Hanchate, 2000 (Dyson and Hanchate, 2000)	50th round NSSO consumer expenditure	Rural/urban Regions &states	Modified trend analysis	7 day dietary recall period improves quantities of consumption in comparison to 30 day period	Average growth rates of per capita consumption over various NSSO rounds from 1972-1994	Cereals Pulses Vegetables Fruit Milk & milk products Meat	1993/94	Not stated	2020
Ganesh-Kumar et al., 2012 (Ganesh-Kumar et al., 2012)	61st round NSSO consumer expenditure	No	QUAIDS model	The relationship between growth in consumption and increasing income stays constant between baseline and projection year	Expenditure elasticities Per capita income growth Population growth	Rice Wheat Pulses Edible Oil Milk Vegetables Sugar Eggs Fish, chicken & meat	2004/05	4% 5% 6%	2020/21 2025/26
Kumar et al., 2009 (Kumar et al., 2009)	61st round NSSO consumer expenditure	Rural/urban	Food characteristic demand system	Per capita expenditure used as a proxy for income Urban income growth rate 3 times that of rural population	Per capita income growth Population growth Urbanisation Region Income group	Rice Wheat Coarse cereals Pulses Total cereals Foodgrains	2004/05	7.91%	2021/22
OECD-FAO 2017 (OECD-FAO, 2017)	OECD and FAO databases	No	Economic partial equilibrium model based on Aglink and Cosimo models	Constant real exchange rate Competitive world markets for agricultural commodities	Population growth Consumer prices Producer prices	Wheat Maize Coarse grains Rice Soybean Oilseed Vegetable oil Sugar Meat Dairy Fish & seafood	2014–16	8.1%	2026
Rosegrant et al., 1999 (Rosegrant et al., 1999)	FAO food balance sheets	No	Updated partial-equilibrium global IMPACT model	The demand elasticity structure assumes that the expected rise in per capita income, commercialisation and urbanisation will cause a shift from main staples to high-value products, for example live-stock products.	Income elasticities Per capita income growth Population growth Effective consumer price Feed ratio Feed efficiency Effective feed price Commodity index specific for livestock	Wheat Maize Rice All cereals Other grains Meat	1993	Not stated	2020

GDP, gross domestic product; FBS, food balance sheet; HHCE, household consumption expenditure; NSSO, National Sample Survey Office; USDA PSD, United States Department of Agriculture production, supply and distribution; FAO, Food and Agriculture Organisation of the United Nations; FAPRI, Food and Agricultural Policy Research Institute; QUAIDS, quadratic almost ideal demand system; OECD, Organisation for Economic Co-operation and Development; IMPACT, international model for policy analysis of agricultural commodities and trade.

The projection methods used by the included studies were heterogeneous. One study used modified trend analysis of prior NSSO collection rounds spanning 22 years (Dyson and Hanchate, 2000). Six studies used projection methods based on a variety of socio-economic demand characteristics such as population growth and urbanisation, using baseline data collected by the NSSO (Amarasinghe et al., 2007; Bhalla et al., 1999; Chand, 2007; Dastagiri, 2004; Ganesh-Kumar et al., 2012; Kumar et al., 2009). Of these, one study used the food characteristic demand system (FCDS) to derive demand elasticities taking into account regional effects and income distribution (Kumar et al., 2009), one study used the quadratic almost ideal demand system (QUAIDS) method (Ganesh-Kumar et al., 2012), and one used the generalised least square (GLS) procedure (Dastagiri, 2004) to calculate demand or expenditure elasticities. These were then incorporated into models with other variables of demand, most frequently population growth, income growth and urbanisation.

Three studies employed econometric partial equilibrium projection models; either the international model for policy analysis of agricultural commodities and trade (IMPACT) model developed by IFPRI (Rosegrant et al., 1999) or commodity outlook models (Carriquiry et al., 2010;OECD-FAO, 2017). Partial equilibrium models incorporate demand (consumption), a simplified representation of supply (production), and trade within the agricultural sector, considered in isolation from other economic sectors (Parappurathu et al., 2014). The partial equilibirium models included in this review are global and are used to generate multi-regional projections. The IMPACT model incorporates variables such as urbanisation, population growth, commodity prices and growth in agricultural productivity, as well as global trade policy. It assumes that income growth will cause significant shifts from main staples to meat and livestock products, mostly in low and middle income countries (Rosegrant et al., 1995). The Food and Agricultural Policy Research Institute (FAPRI) Agricultural Outlook model uses a previously developed macroeconomic forecast and assumes existing farm policy and current trade agreements and custom unions (Carriquiry et al., 2010). The OECD-FAO Agricultural Outlook model uses macroeconomic forecasts of the OECD Economic Outlook and International Monetary Fund (IMF) World Economic Outlook, and also assumes current agricultural policies remain unchanged (OECD-FAO, 2017). The partial equilibrium models use national level food availability data as opposed to NSSO data. One study derives consumption projections of food groups from proportional share of future per capita dietary energy intake based on consumer expenditure projections (Alexandratos and Bruinsma, 2012).

### 3.2 Study reporting quality

We evaluated each study for six features of reporting quality (Table 3). Most studies reported projection timeline and baseline data source. Few studies reported limitations of the projection methods used, and validation of the models. One study met all six quality criteria (OECD-FAO, 2017), and one study met five criteria (Ganesh-Kumar et al., 2012) (supplementary material S2). No relevant study was excluded from the review based on reporting quality.

**Table 3:** Number of studies meeting reporting quality criteria (n = 11).

#	Quality Indicator Description	Number of studies
**1**	Baseline data source stated	10
**2**	Clear description of projections methodology	8
**3**	Validation of projections methodology reported	2
**4**	Explanation of assumptions and variables	9
**5**	Clearly stated projections timeline	11
**6**	Acknowledgement of limitations of projections methods	1

### 3.3 Future dietary trends at national level in India

**Fig. 2:**
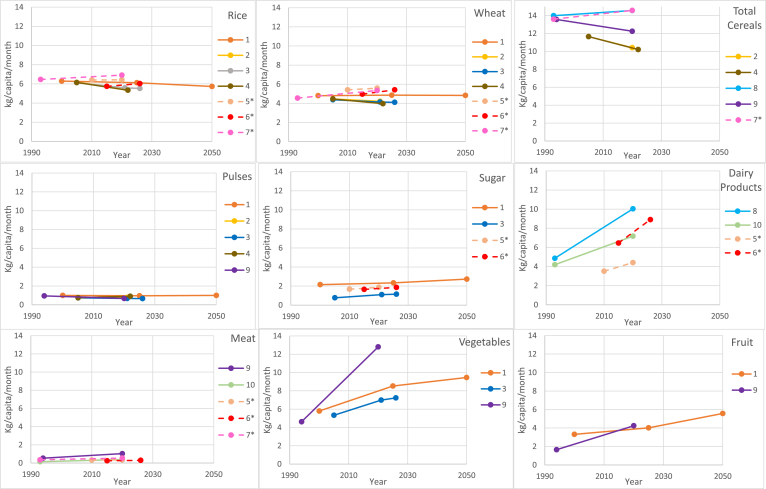
**Overview of future direct consumption trends in India from selected studies in the review.** Legend numbers correspond to reference numbers of the studies. *consumption data not obtained from NSSO.

Baseline and projected consumption data were available for 9 foods or food groups reported on two or more occasions. Per capita consumption of rice (n = 7 studies), wheat (n = 7 studies) and pulses (n = 5 studies) is projected to remain broadly unchanged in the future at around 6kg, 5kg and 1kg/capita/month, respectively (Fig. 2; supplementary material S3 for raw data). Total cereal consumption (inclusive of rice, wheat, maize, sorghum and millet; n = 5 studies) is projected to decline in three studies using NSSO consumption data with negative price elasticities for cereals (Chand, 2007; Dyson and Hanchate, 2000; Kumar et al., 2009) and increase in one study using NSSO consumption data with positive price elasticies for cereals (James et al., 2016). One study that used the IMPACT model projected a slight increase in future total cereal consumption (Rosegrant et al., 1999). Per capita consumption of all other foods or food groups, namely sugar, dairy, meat, vegetables and fruit, is projected to increase in the future in all studies irrespective of data source or projection method, with the magnitude of future increase greatest for dairy and vegetables. In one study reporting values in kcal/capita that could not be presented graphically, findings are consistent with those of other studies: namely a decrease in future cereal consumption and an increase in dairy, sugar and meat consumption by 2050 (Alexandratos and Bruinsma, 2012).

Three studies examined the effect of differing GDP growth scenarios on future consumption of selected food groups. Compared to scenarios with conservative GDP growth, future consumption of dairy (n = 2) and meat (n = 1) is projected to be greater in scenarios with higher GDP growth rate (Bhalla et al., 1999; Dastagiri, 2004). However, projected consumption of rice, wheat, pulses, sugar and vegetables (n = 1) are predicted to remain relatively unchanged irrespective of GDP growth scenarios (Ganesh-Kumar et al., 2012). Disaggregation of consumption projections into urban and rural populations (n = 5 studies) suggested that different levels of consumption of some foods at baseline would remain in the future: cereal consumption is lower (Chand, 2007; Dyson and Hanchate, 2000; Kumar et al., 2009) and fruit consumption is higher (Amarasinghe et al., 2007; Dyson and Hanchate, 2000) in urban settings. These studies provided little consistent evidence from projections that these urban-rural differences in consumption would differ in the future (supplementary material S4).

## 4 Discussion

**Fig. 3:**
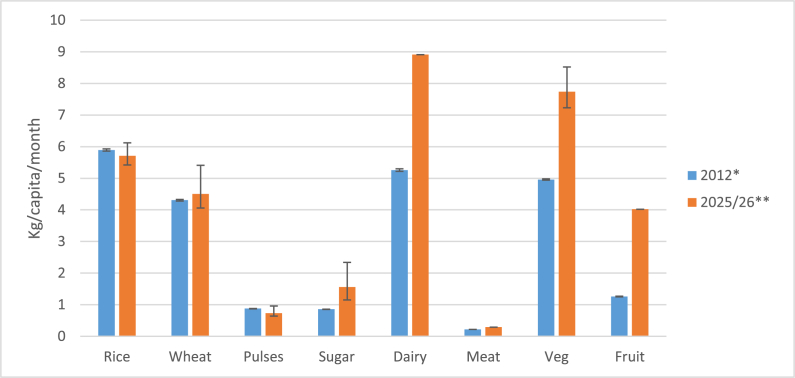
**Comparison of diets in India in 2012 and projected diets in 2025**–26.*2012 values are mean consumption for adults aged 16–59 years calculated from results of 68th NSSO round (25). Error bars represent 95% confidence interval. **2025–26 consumption represents the mean of selected of studies included in this review that project to this time point. Error bars represent upper and lower range of the data where more than one value available for each food group.

We systematically reviewed the available published and grey literature to identify projection studies of future food consumption in India. Despite heterogeneous methodological approaches and study quality, several consistent features emerge in the identified papers. First, per capita cereal and pulse consumption is projected to remain relatively constant even in long-term projections to 2050 irrespective of income growth assumptions. Second, per capita consumption of sugar, dairy, meat, vegetables and fruit is projected to increase, with the magnitude of the projected increase in meat and dairy consumption expected to be directly related to income growth. Third, in urban areas, current and future consumption of cereals is lower and fruit is higher than in rural areas. To summarise these projected changes we compared reported consumption from 2012 (Aleksandrowicz et al., 2017) with average consumption projected in 3 studies (Amarasinghe et al., 2007; Ganesh-Kumar et al., 2012;OECD-FAO, 2017) that provided estimates for 2025 or 2026 (Fig. 3 - data are available for 8 food groups). Whilst differences in methodology do not allow for exact comparison, the figure confirms substantial differences in absolute projected per capita consumption of vegetables, fruits, dairy and sugar.

### 4.1 Comparison with other literature

The trends in projected future Indian diets found by this review are in line with the global “nutrition transition” towards diets with greater consumption of animal products and processed foods high in salt and refined sugar (Drewnowski and Popkin, 1997). Similar trends have been reported in several countries including China which, during the latter two decades of the 20th Century when rates of annual per capita income growth were high, experienced a 30% increase in average consumption of animal products across all income levels (Du et al., 2004). Currently, a large proportion of the Indian population consumes a lacto-ovo vegetarian diet, however the evidence presented here suggests that the consumption of animal products will rapidly rise with growing incomes. Unless cultural practices change, it is likely that the increase in animal products in India will largely relate to dairy consumption.

Global trends in increased consumption of animal products and processed foods have typically occurred alongside a reduction in cereal consumption and increased consumption of other food groups (Rosegrant et al., 1999). These trends are also reflected in the findings of our review, which indicates stagnation or potential decline in total cereal consumption in India, alongside increased consumption of fruit and vegetables. Fruit and vegetables are vital sources of micronutrients, and an increase in their consumption would potentially lead to increased diversity of micronutrient intake with concomitant health benefits (Wang et al., 2014), an encouraging finding against the backdrop of India's high micronutrient deficiency rates. However, it should be noted that a recent analysis of NSSO consumer expenditure data found that when disaggregated into items within each food group, a large proportion of vegetable intake was accounted for by potatoes, and similarly for fruit the largest proportional share was from bananas (Minocha et al., 2018). The increased fruit and vegetable consumption suggested may therefore not directly reflect increased diversity of diets or micronutrient intake.

Diverse diets comprising a variety of food groups including meat and dairy have been advocated in the national food based dietary guidelines for India that were most recently updated in 2011 (National Institute of Nut, 2011). In keeping with these recommendations our findings suggest increased consumption of a number of food groups. Although cereal consumption appears to decline, it is predicted to remain the most consumed food group per capita, which accords with recommendations for diets high in complex carbohydrates such as cereals (National Institute of Nut, 2011). However, the majority of the cereals consumed in India are likely to be refined rice and wheat, rather than more nutritious coarse cereals (Rao et al., 2018), and diets in reality may therefore not consistently align with recommendations for dietary intake of fibre and complex carbohydrates.

The association of diets rich in fruit, vegetables and plant-based unrefined carbohydrates such as cereals and pulses with positive health outcomes (particularly NCDs) have been well established (Segovia-Siapco and Sabaté, 2018; Petersen et al., 2017); there is also now accumulating evidence to suggest these dietary patterns are more environmentally sustainable in comparison to diets higher in animal products (Segovia-Siapco and Sabaté, 2018; Springmann et al., 2016; Aleksandrowicz et al., 2016). Public health policy makers therefore face the challenge of addressing micronutrient deficiencies by recommending increasing dietary diversity within the context of what is available and affordable, whilst trying to ensure this minimises risk to the environment or to chronic disease development.

### 4.2 Strengths and limitations

This review has several strengths including our thorough and systematic approach to identify all relevant studies in published and grey literature, to provide an overview of the available projections of food consumption in India. Previous research on dietary projections has focused on staple cereals but despite heterogeneity in methods, data and reporting quality of the included studies we were able to report projections of future consumption for a diverse range of foods and food groups.

There are a number of limitations to our review. Firstly, we searched for studies reported in the English language only, and may have missed studies published in other languages, particularly other official languages of India. Second, the variation in the projection methods and the gaps in reporting of the studies included in the review limited our ability to conduct quantitative analyses. Third, our review was able to report projections for nine major food groups, but sufficient data were not available to report projections of other foods such as salt, oils and nuts/seeds that have been linked to both population health and the environment. Additionally, given the nature of the data available, our review was not able to report and adjust for projected trends in total dietary energy intake. We are therefore not able to estimate directly the effects of projected changes to food consumption on health. Some gaps therefore remain in our understanding of the likely future composition and health effects of diets in India.

There are also limitations to consider arising from the sources of data used in projection studies. The majority of the studies used household consumer expenditure data collected by the NSSO in India, either from a single data collection round or multiple rounds over time. Household consumer expenditure surveys have many advantages over other methods of collecting consumption information (Fiedler et al., 2012), and the NSSO database is considered a high-quality nationally representative data source (Natrajan and Jacob, 2018). However as collection relies on self-reporting of quantities and monthly expenditure on different food items by participants, it is subject to recall bias and misreporting, particularly of culturally sensitive foods (Natrajan and Jacob, 2018). Additionally, as expenditure is used as a proxy for consumption, household food waste, sharing of food between households, and meals eaten outside the home are not consistently included in reports using NSSO data, making comparisons between rounds difficult (Fiedler et al., 2012). Several studies analysed data from FAO food balance sheets and used data on national-level food availability as a proxy for household consumption. Findings of this review should therefore be interpreted as projections of relative, rather than absolute, future changes in consumption.

In most cases, the models used to estimate future food consumption patterns were parameterised to account for likely future changes in demand-side factors such as population growth, urbanisation, expenditure elasticities and income growth. However, it is possible that food supply may become a limiting factor according to predicted impacts of environmental change on cereal and vegetable yields (Knox et al., 2012, 2016; Scheelbeek et al., 2018), and future consumption projections would need to take this into consideration. The IMPACT model has recently been updated to incorporate food security and climate change scenarios (Robinson et al., 2015). Lastly, India is a very culturally diverse setting, with food choices influenced by household purchasing power and cultural factors such as caste and religion (Natrajan and Jacob, 2018). Future consumption projections may seek to capture cultural differences by disaggregating projections into states or including population sub-groups into models to reflect the contextual relationships that drive dietary choices.

### 4.3 Future possibilities and policy relevance

Our review identified a relatively small number of studies projecting future diets in India using a diversity of approaches. There would be a benefit to developing a set of best practices for dietary projection work to help researchers and policy makers. Methods for the validation of projection approaches (including defined sensitivity analysis) should also be considered. Similarly, while interdisciplinary systematic reviews are increasingly performed (Aleksandrowicz et al., 2016; Scheelbeek et al., 2018), current review guidelines are tailored to biomedical sciences. There is a need to develop robust interdisciplinary systematic review methods and quality assessment criteria, to improve scientific reporting in academic literature.

The projected changes in food consumption in India identified in this review may have important impacts on population health, potentially for both the burdens of undernutrition and non-communicable disease, but the changes will require a food system that is able to deliver these new diets in an equitable and environmentally-sustainable manner. Income growth and urbanisation are currently major drivers of the demand for food, and there are major implications for the environment from food production in India (Green et al., 2018; Harris et al., 2017; Vetter et al., 2017). Comprehensive improvement of food systems, on nutritional, environmental, and social fronts, will require a range of policies that focus on food security, sustainable nutrition and healthy dietary choices.

Beyond the benefit of predicting supply-demand gaps in the future, consumption projections provide a means to determine the impacts of changing consumption patterns on environmental outcomes such as land and water use, and health. This will be useful for policymakers across health, nutrition, agriculture and environmental sectors to respond to the changing reality of their country, thus determining the future health of their population.

## Declarations of interest

None.

## References

[bib3] Aleksandrowicz L., Green R., Joy E.J.M., Smith P., Haines A. (2016). The impacts of dietary change on greenhouse gas emissions, land use, water use, and health: a systematic review. PLoS One.

[bib4] Aleksandrowicz L., Tak M., Green R., Kinra S., Haines A. (2017). Comparison of food consumption in Indian adults between national and sub-national dietary data sources. Br. J. Nutr..

[bib5] Alexandratos N., Bruinsma J. (2012). World Agriculture towards 2030/2050: the 2012 Revision. ESA Working Paper No. 12-03.

[bib6] Amarasinghe U.A., Shah T., Singh O.P. (2007). Changing Consumption Patterns: Implications on Food and Water Demand in India.

[bib7] Bhalla G.S., Hazell P., Kerr J. (1999). Prospects for India's Cereal Supply and Demand to 2020.

[bib8] Carriquiry M., Dong F., Du X., Elobeid A.E., Fabiosa J.F., Hart C. (2010). FAPRI 2010 U.S. And World Agricultural Outlook. FAPRI Staff Reports No 4.

[bib9] Chand R. (2007). Demand for foodgrains. Econ. Pol. Wkly..

[bib10] Dastagiri M.B. (2004). Demand and Supply Projections for Livestock Products in India.

[bib11] Drewnowski A., Popkin B.M. (1997). The nutrition transition: new trends in the global diet. Nutr. Rev..

[bib12] Du S., Mroz T.A., Zhai F., Popkin B.M. (2004). Rapid income growth adversely affects diet quality in China - particularly for the poor!. Soc. Sci. Med..

[bib13] Dyson T., Hanchate A. (2000). India's demographic and food prospects: state-level analysis. Econ. Pol. Wkly..

[bib14] Fiedler J.L., Lividini K., Bermudez O.I., Smitz M.F. (2012). Household Consumption and Expenditures Surveys (HCES): a primer for food and nutrition analysts in low-and middle-income countries. Food Nutr. Bull..

[bib15] Foley J.A., DeFries R., Asner G.P., Barford C., Bonan G., Carpenter S.R. (2005). Global consequences of land use. Science.

[bib16] Ganesh-Kumar A., Mehta R., Pullabhotla H., Prasad S.K., Ganguly K., Gulati A. (2012). Demand and Supply of Cereals in India.

[bib17] Government of India (2015). India's Intended Nationally Determined Contribution: Working towards Climate Justice. http://www4.unfccc.int/ndcregistry/PublishedDocuments/India%20First/INDIA%20INDC%20TO%20UNFCCC.pdf.

[bib18] Green R.F., Joy E.J.M., Harris F., Agrawal S., Aleksandrowicz L., Hillier J. (2018). Greenhouse gas emissions and water footprints of typical dietary patterns in India. Sci. Total Environ..

[bib19] Grieger J.A., Johnson B.J., Wycherley T.P., Golley R.K. (2017). Evaluation of simulation models that estimate the effect of dietary strategies on nutritional intake: a systematic review. J. Nutr..

[bib20] Harris F., Green R.F., Joy E.J.M., Kayatz B., Haines A., Dangour A.D. (2017). The water use of Indian diets and socio-demographic factors related to dietary blue water footprint. Sci. Total Environ..

[bib21] (2010). Indian Network for Climate Change Assessment. India: Greenhouse Gas Emissions 2007.

[bib22] James K.L., Randall N.P., Haddaway N.R. (2016). A methodology for systematic mapping in environmental sciences. Environ. Evid..

[bib23] Knox J., Hess T., Daccache A., Wheeler T. (2012). Climate change impacts on crop productivity in Africa and South Asia. Environ. Res. Lett..

[bib24] Knox J., Daccache A., Hess T., Haro D. (2016). Meta-analysis of climate impacts and uncertainty on crop yields in Europe. Environ. Res. Lett..

[bib25] Kumar P., Joshi P.K., Birthal P.S. (2009). Demand projections for food grains in India. Agric. Econ. Res. Rev..

[bib26] Liberati A., Altman D.G., Tetzlaff J., Mulrow C., Gøtzsche P.C., Ioannidis J.P.A. (2009). The PRISMA statement for reporting systematic reviews and meta-analyses of studies that evaluate health care interventions: explanation and elaboration. PLoS Med..

[bib27] Minocha S., Thomas T., Kurpad A.V. (2018). Are ‘fruits and vegetables’ intake really what they seem in India?. Eur. J. Clin. Nutr..

[bib28] Misra A., Singhal N., Sivakumar B., Bhagat N., Jaiswal A., Khurana L. (2011). Nutrition transition in India: secular trends in dietary intake and their relationship to diet‐related non‐communicable diseases. J. Diabetes.

[bib29] National Institute of Nutrition (2011). Dietary Guidelines for Indians: a Manual.

[bib30] Natrajan B., Jacob S. (2018). ‘Provincialising’Vegetarianism. Economic & Political Weekly.

[bib2] OECD/FAO (2017). OECD-FAO Agricultural Outlook 2017-2026.

[bib31] Parappurathu S., Kumar A., Kumar S., Jain R. (2014). Commodity Outlook on Major Cereals in India.

[bib32] Petersen K.S., Flock M.R., Richter C.K., Mukherjea R., Slavin J.L., Kris-Etherton P.M. (2017). Healthy dietary patterns for preventing cardiometabolic disease: the role of plant-based foods and animal products. Current developments in nutrition.

[bib33] Rao N.D., Min J., DeFries R., Ghosh-Jerath S., Valin H., Fanzo J. (2018). Healthy, affordable and climate-friendly diets in India. Glob. Environ. Chang..

[bib34] Robinson S., Mason-D'Croz D., Sulser T., Islam S., Robertson R., Zhu T. (2015). The International Model for Policy Analysis of Agricultural Commodities and Trade (IMPACT): Model Description for Version 3. IFPRI Discussion Paper 1483.

[bib35] Rodell M., Velicogna I., Famiglietti J.S. (2009). Satellite-based estimates of groundwater depletion in India. Nature.

[bib36] Rosegrant M.W., Agcaoili-Sombilla M., Perez N.D. (1995). Global Food Projections to 2020: Implications for Investment.

[bib37] Rosegrant M.W., Leach N., Gerpacio R.V. (1999). Alternative futures for world cereal and meat consumption. Proc. Nutr. Soc..

[bib38] Scheelbeek P.F.D., Bird F.A., Tuomisto H.L., Green R., Harris F.B., Joy E.J.M. (2018). Effect of environmental changes on vegetable and legume yields and nutritional quality. Proc. Natl. Acad. Sci. Unit. States Am..

[bib39] Segovia-Siapco G., Sabaté J. (2018). Health and sustainability outcomes of vegetarian dietary patterns: a revisit of the EPIC-Oxford and the Adventist Health Study-2 cohorts. Eur. J. Clin. Nutr..

[bib40] Springmann M., Godfray H.C.J., Rayner M., Scarborough P. (2016). Analysis and valuation of the health and climate change cobenefits of dietary change. Proc. Natl. Acad. Sci. Unit. States Am..

[bib41] (2016). FAO. AQUASTAT Website.

[bib42] Tyndall J. (2010). AACODS Checklist. http://dspace.flinders.edu.au/dspace/.

[bib1] UNICEF-World Health Organization-The World Bank (2017). Joint Child Malnutrition Estimates—Levels and Trends.

[bib43] United Nations. Population Division World Population Prospects 2017. https://esa.un.org/unpd/wpp.

[bib44] Vetter S.H., Sapkota T.B., Hillier J., Stirling C.M., Macdiarmid J.I., Aleksandrowicz L. (2017). Greenhouse gas emissions from agricultural food production to supply Indian diets: implications for climate change mitigation. Agric. Ecosyst. Environ..

[bib45] Wang X., Ouyang Y., Liu J., Zhu M., Zhao G., Bao W. (2014). Fruit and vegetable consumption and mortality from all causes, cardiovascular disease, and cancer: systematic review and dose-response meta-analysis of prospective cohort studies. BMJ.

[bib46] World Health Organization (2003). Globalization, Diets and Noncommunicable Diseases.

[bib47] World Health Organization (2017). Noncommunicable Diseases Progress Monitor 2017.

