# Supplementary material for: Future diets in India: A systematic review of food consumption projection studies
Source: Glob Food Sec. 2019 Dec;23:182–90. doi: 10.1016/j.gfs.2019.05.006 (PMC7212791; doi:10.1016/j.gfs.2019.05.006)
Supplement: Multimedia component 1 [file mmc1.docx]

# Supplementary Information

## S1. Database Search Strategies.

## EMBASE

1. (food OR diet OR nutri* OR consum* or nourish*)

2. ((project* adj3 diet*) OR (future adj3 diet*) OR (trend* adj3 diet*) OR (transition* adj3 diet*) OR (change* adj3 diet*) OR (predict* adj3 diet*) OR (forecast adj3 diet*) OR (prognos* adj3 diet*))

3. (india* OR asia* or "south asia*" OR global)

## Global Health

1. (food OR diet OR nutri* OR consum* or nourish*)

2. ((project* adj3 diet*) OR (future adj3 diet*) OR (trend* adj3 diet*) OR (transition* adj3 diet*) OR (change* adj3 diet*) OR (predict* adj3 diet*) OR (forecast adj3 diet*) OR (prognos* adj3 diet*))

3. (india* OR asia* or "south asia*" OR global)

## MEDLINE

1. (food OR diet OR nutri* OR consum* or nourish*)

2. ((projection adj3 diet*) OR (future adj3 diet*) OR (trend* adj3 diet*) OR (transition* adj3 diet*) OR (change* adj3 diet*) OR (predict* adj3 diet*) OR (forecast adj3 diet*) OR (prognos* adj3 diet*))

3. (india* OR asia* OR "south asia*" OR global)

## PubMed

1. (food OR diet OR nutri* OR consum* or nourish*)

2. ((project* n3 diet*) OR (future n3 diet*) OR (trend* n3 diet*) OR (transition* n3 diet*) OR (change* n3 diet*) OR (predict* n3 diet*) OR (forecast n3 diet*) OR (prognos* n3 diet*))

3. (india* OR asia* or "south asia*" OR global)

## Scopus

1. TITLE-ABS-KEY((food OR diet OR nutri* OR consum* or nourish*)

2. ((project* W/1 diet*) OR (future W/1 diet*) OR (trend* W/1 diet*) OR (transition* W/1 diet*) OR (change* W/1 diet*) OR (predict* W/1 diet*) OR (forecast W/1 diet*) OR (prognos* W/1 diet*))

3. (india* OR asia* or "south asia*" OR global)

## Web of Science

1. TS=((food OR diet OR nutri* OR consum* or nourish*)

2. ((project* NEAR/1 diet*) OR (future NEAR/1 diet*) OR (trend* NEAR/1 diet*) OR (transition* NEAR/1 diet*) OR (diet* NEAR/1 change) OR (predict* NEAR/1 diet*) OR (forecast NEAR/1 diet*) OR (prognos* NEAR/1 diet*))

3. (india* OR asia* or "south asia*" OR global)

## S2. Reporting quality of studies included in the review

| Author & Year | Baseline Data Source Stated | Clear Description of Projections Methods | Validation of Methods Reported | Explanation of Assumptions & Variables | Clearly Stated Projections Timeline | Acknowledgment of Limitations of Method | Total |
| --- | --- | --- | --- | --- | --- | --- | --- |
| Alexandratos & Bruinsma 2012 (FAO) (1) | 1 | 1 | 0 | 0 | 1 | 0 | 3 |
| Amarasinghe et al 2007 (2) | 1 | 1 | 0 | 1 | 1 | 0 | 4 |
| Bhalla et al 1999 (3) | 1 | 0 | 0 | 1 | 1 | 0 | 3 |
| Carriquiry et al 2010 (FAPRI) (4) | 1 | 0 | 0 | 1 | 1 | 0 | 3 |
| Chand 2007  (5) | 1 | 0 | 0 | 1 | 1 | 0 | 3 |
| Dastagiri 2004 (6) | 1 | 1 | 0 | 1 | 1 | 0 | 4 |
| Dyson & Hanchate 2000 (7) | 1 | 1 | 0 | 1 | 1 | 0 | 4 |
| Ganesh-Kumar et al 2012 (8) | 1 | 1 | 1 | 1 | 1 | 0 | 5 |
| Kumar et al 2009 (9) | 1 | 1 | 0 | 1 | 1 | 0 | 4 |
| OECD-FAO 2017 (10) | 1 | 1 | 1 | 1 | 1 | 1 | 6 |
| Rosegrant et al 1999 (11) | 0 | 1 | 0 | 0 | 1 | 0 | 2 |

## S3. Baseline and projected food or food group consumption figures from studies included in the systematic review

| Author & Year | Food groups | Baseline Year | Baseline Food consumption | GDP Scenarios | Projection Year | Projected Food Consumption | Projection Year | Projected Food Consumption |
| --- | --- | --- | --- | --- | --- | --- | --- | --- |
| Alexandratos & Bruinsma 2012 (FAO) (1) | Cereals Milk & dairy Sugar (raw) Meat | 2005/07 | 151 kcal/capita/day  67 kcal/capita/day  18.8 kcal/capita/day  3.1 kcal/capita/day | 4.4% | 2050 | 144 kcal/capita/day  110 kcal/capita/day  29 kcal/capita/day  18.3 kcal/capita/day |  |  |
| Amarasinghe et al 2007 (2) | Rice Wheat Pulses Vegetables Fruits Sugar | 2000 | 6.29 kg/capita/month  4.80 kg/capita/month  0.99 kg/capita/month  5.79 kg/capita/month  3.31 kg/capita/month  2.15 kg/capita/month | Not stated | 2025 | 6.12 kg/capita/month  4.86 kg/capita/month  0.96 kg/capita/month  8.52 kg/capita/month  4.02 kg/capita/month  2.34 kg/capita/month | 2050 | 5.73 kg/capita/month  4.83 kg/capita/month  1.00 kg/capita/month  9.46 kg/capita/month  5.57 kg/capita/month  2.73 kg/capita/month |
| Bhalla et al 1999 (3) | Cereals Milk & milk products | 1993 | 13.99 kg/capita/month  4.87 kg/capita/month | 2% | 2020 | 14.57 kg/capita/month  10.04 kg/capita/month |  |  |
|  | Cereals Milk & milk products |  | 13.99 kg/capita/month  4.87 kg/capita/month | 3.7% |  | 15.51 kg/capita/month  18.25 kg/capita/month |  |  |
|  | Cereals Milk & milk products |  | 13.99 kg/capita/month  4.87 kg/capita/month | 6% |  | 16.73 kg/capita/month  40.45 kg/capita/month |  |  |
| Carriquiry et al 2010 (FAPRI) (4) | Wheat Rice Sugar Meat Dairy | 2009/10 | 5.42 kg/capita/month  6.40 kg/capita/month  1.67 kg/capita/month  0.33 kg/capita/month  3.52 kg/capita/month | Not stated | 2019/2020 | 5.58 kg/capita/month  6.43 kg/capita/month  1.87 kg/capita/month  0.35 kg/capita/month  4.42 kg/capita/month |  |  |
| Chand 2007 (5) | Rice Wheat Pulses  Total cereals | 2004/05 | 6.15 kg/capita/month  4.46 kg/capita/month  1.05 kg/capita/month  11.66 kg/capita/month | 9% | 2020/21 | 5.57 kg/capita/month  4.22 kg/capita/month  0.63 kg/capita/month  10.42 kg/capita/month |  |  |
| Dastagiri 2004 (6) | Milk Meat | 1993 | 4.19 kg/capita/month  0.14 kg/capita/month | 4% | 2020 | 7.18 kg/capita/month  0.39 kg/capita/month |  |  |
|  | Milk Meat |  | 4.19 kg/capita/month  0.14 kg/capita/month | 5% |  | 9.14 kg/capita/month  0.91 kg/capita/month |  |  |
|  | Milk Meat |  | 4.19 kg/capita/month  0.14 kg/capita/month | 7% |  | 14.10 kg/capita/month  3.12 kg/capita/month |  |  |
| Dyson & Hanchate 2000 (7) | Cereals Pulses Vegetables Fruit Meat | 1993/94 | 13.57 kg/capita/month  0.95 kg/capita/month  4.62 kg/capita/month  1.65 kg/capita/month  0.53 kg/capita/month | Not stated | 2020 | 12.26 kg/capita/month  0.68 kg/capita/month  12.80 kg/capita/month  4.24 kg/capita/month  1.03 kg/capita/month |  |  |
| Ganesh-Kumar et al 2012 (8) | Rice Wheat Pulses Vegetables Sugar | 2004/05 | 6.12 kg/capita/month  4.38 kg/capita/month  0.74 kg/capita/month  5.33 kg/capita/month  0.77 kg/capita/month | 4% | 2020/21 | 5.59 kg/capita/month  4.14 kg/capita/month  0.67 kg/capita/month  6.98 kg/capita/month  1.10 kg/capita/month | 2025/26 | 5.53 kg/capita/month  4.11 kg/capita/month  0.66 kg/capita/month  7.23 kg/capita/month  1.15 kg/capita/month |
|  | Rice Wheat Pulses Vegetables Sugar |  | 6.12 kg/capita/month  4.38 kg/capita/month  0.74 kg/capita/month  5.33 kg/capita/month  0.77 kg/capita/month | 5% |  | 5.55 kg/capita/month  4.12 kg/capita/month  0.66 kg/capita/month  7.16 kg/capita/month  1.13 kg/capita/month |  | 5.46 kg/capita/month  4.08 kg/capita/month  0.65 kg/capita/month  7.51 kg/capita/month  1.21 kg/capita/month |
|  | Rice Wheat Pulses Vegetables Sugar |  | 6.12 kg/capita/month  4.38 kg/capita/month  0.74 kg/capita/month  5.33 kg/capita/month  0.77 kg/capita/month | 6% |  | 5.50 kg/capita/month  4.10 kg/capita/month  0.65 kg/capita/month  7.34 kg/capita/month  1.17 kg/capita/month |  | 5.42 kg/capita/month  4.06 kg/capita/month  0.64 kg/capita/month  7.68 kg/capita/month  1.24 kg/capita/month |
| Kumar et al 2009 (9) | Rice Wheat Pulses  Total cereals | 2004/05 | 6.15 kg/capita/month  4.46 kg/capita/month  0.75 kg/capita/month  11.66 kg/capita/month | 7.91% | 2021/22 | 5.34 kg/capita/month  3.97 kg/capita/month  0.91 kg/capita/month  10.22 kg/capita/month |  |  |
| OECD-FAO 2017 (10) | Wheat  Rice  Sugar  Meat  Dairy | 2014-16 | 4.97 kg/capita/month  5.74 kg/capita/month  1.65 kg/capita/month  0.25 kg/capita/month  6.46 kg/capita/month | 8.1% | 2026 | 5.41 kg/capita/month  6.03 kg/capita/month  1.86 kg/capita/month  0.29 kg/capita/month  8.91 kg/capita/month |  |  |
| Rosegrant et al 1999 (11) | Wheat Rice  Total cereals Meat | 1993 | 4.55 kg/capita/month  6.46 kg/capita/month  13.60 kg/capita/month  0.36 kg/capita/month | Not stated | 2020 | 5.34 kg/capita/month  6.91 kg/capita/month  14.58 kg/capita/month  0.54 kg/capita/month |  |  |

## S4. Summary of rural and urban trends in future direct consumption in India from selected studies in the review.

## Legend numbers correspond to reference numbers of the studies.


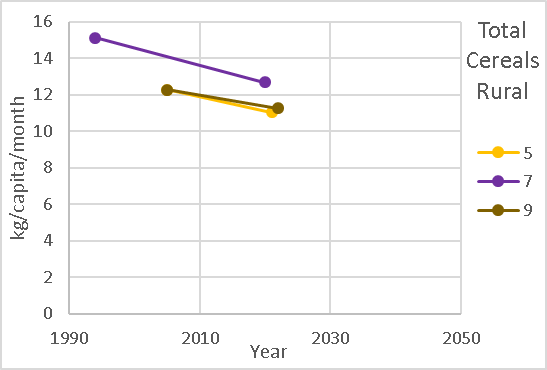

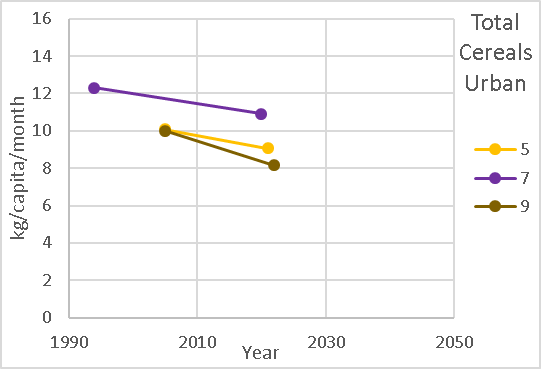


# References

1. Alexandratos N, Bruinsma J. World Agriculture Towards 2030/2050: The 2012 Revision. ESA Working Paper No. 12-03. Rome: FAO; 2012.

2. Amarasinghe UA, Shah T, Singh OP. Changing consumption patterns: Implications on food and water demand in India. Columbo, Sri Lanka: International Water Management Institute; 2007.

3. Bhalla GS, Hazell P, Kerr J. Prospects for India's cereal supply and demand to 2020. Washington D.C., USA: International Food Policy Research Institute; 1999.

4. Carriquiry M, Dong F, Du X, Elobeid AE, Fabiosa JF, Hart C, et al. FAPRI 2010 U.S. and World Agricultural Outlook. FAPRI Staff Reports No 4. IOWA, USA: FAPRI; 2010.

5. Chand R. Demand for foodgrains. Economic and Political Weekly. 2007;42(52):10-3.

6. Dastagiri MB. Demand and supply projections for livestock products in India. New Delhi, India: National Centre for Agricultural Economics and Policy Research; 2004.

7. Dyson T, Hanchate A. India's demographic and food prospects: state-level analysis. Economic and Political Weekly. 2000;35(46):4021-36.

8. Ganesh-Kumar A, Mehta R, Pullabhotla H, Prasad SK, Ganguly K, Gulati A. Demand and supply of cereals in India. New Delhi, India: International Food Policy Research Institute; 2012.

9. Kumar P, Joshi PK, Birthal PS. Demand projections for food grains in India. Agricultural Economics Research Review. 2009;22(2):237-43.

10. OECD/FAO. OECD-FAO Agricultural Outlook 2017-2026. Paris, France: OECD Publishing; 2017.

11. Rosegrant MW, Leach N, Gerpacio RV. Alternative futures for world cereal and meat consumption. Proceedings of the Nutrition Society. 1999;58(2):219-34. <https://doi.org/10.1017/S0029665199000312>.
